# The COVID‐19 pandemic: some lessons learned about crisis preparedness and management, and the need for international benchmarking to reduce deficits

**DOI:** 10.1111/1462-2920.15029

**Published:** 2020-05-03

**Authors:** Kenneth Timmis, Harald Brüssow

**Affiliations:** ^1^ Institute of Microbiology, Technical University of Braunschweig Braunschweig Germany; ^2^ Department of Biosystems Group of Gene Technology, K. U. Leuven Leuven Belgium

If, despite the explicit warning of the World Health Organization in 2011 that ‘The world is ill‐prepared to respond to a severe influenza pandemic or to any similarly global, sustained and threatening public‐health emergency’ (https://apps.who.int/gb/ebwha/pdf_files/WHA64/A64_10-en.pdf), it was not apparent to those in charge, and to the general public—i.e., those suffering from COVID‐19 infections and the funders of health services (tax/insurance payers)—that existing health systems had inherent vulnerabilities which could prove to be devastating when seriously stressed, the SARS‐CoV‐2 pandemic (e.g., see Brüssow, 2020) has brutally exposed it now. In some countries, preparedness, despite being officially considered to be of strong operational readiness against health emergencies (Kandel *et al*., 2020), was inadequate at multiple levels (e.g., Horton, 2020). Similarly, a fundamental lack of preparedness is the case for a number of impending non‐health crises (e.g., global warming, poverty, the soil crisis, etc.). Once we are over the COVID‐19 pandemic, important questions will be: what have we learned/can we learn and how can we improve our systems? Below, we argue for the necessity for major realignment of crisis responsiveness, and indeed of health system operationality, based on international benchmarking and adequately funded preparedness. International benchmarking is mandatory, because it has become clear that there is a wide range of effectiveness in the ability of different countries with developed economies to respond to this crisis (and probably others), and the tax‐paying public has no compelling reason to tolerate perpetuation of factors underlying poor responses to crises.

## Disparity in country/regional responses to SARS‐CoV‐2

### Diagnosis

Leaving aside for the moment decisions about whether to robustly contain the outbreak—to kill it by throttling—the classical strategy of infection control, but which leaves most of the population susceptible to a new outbreak, or attempt to manage an outbreak to achieve herd immunity and a population unsusceptible to a repeat outbreak, it is obviously imperative to know how the outbreak is spreading and how effective are any containment measures that are instituted, so that a change in course of action remains an option. Widespread testing for the viral pathogen, with correct sampling and analysis procedures, is thus essential. This enables, *inter alia*, calculation of reliable mortality (case fatality) rates,^1^ which ordinarily play a key role in determining crisis response policy, calculation of the Basic Reproduction Number, *R*_0_, and reliable modelling of transmission and mortality trends. There have been enormous differences in testing coverages among countries. Early predictions of transmission and mortality trends are only as good as the adequacy of the information fed into models so, with inadequate testing, prediction of trends are unreliable. Mortality‐based policy formulation for SARS‐CoV‐2 responses in some countries was based on incomplete information.

- *Diagnostics and widespread testing are the basis of informed policy development for crisis management of infectious disease epi/pandemics and must become its centrepiece*

The disparity of SARS‐CoV‐2 testing coverage in different countries is much discussed but the reasons are sometimes rather opaque. There are many tests available (e.g., see https://www.finddx.org/covid-19/pipeline/), so one bottleneck would appear to be a limitation in authorized testing facilities. Scaling up testing in existing official centres requires not only acquisition of the appropriate instrumentation and reagents, but also of competent personnel disposing of the necessary expertise, which may constitute a bottleneck. However, it is abundantly clear that the instrumentation and expertise needed to carry out SARS‐CoV‐2 testing are widespread in academic research laboratories, as is the eagerness of many research groups to contribute to efforts to combat the pandemic. While issues of safety, quality control, logistics, data reporting and security and so on, need to be addressed, a failure in some countries to harness early in the crisis the expertise and enthusiasm of young researchers to fulfil a key need and, with it, the opportunity to acquire data that could have resulted in responses that saved lives, is regrettable. Paradoxically, while there have been frantic efforts to open new hospital facilities to accommodate COVID‐19 patients, and to recruit the health professionals needed to operate them, there has been an indiscriminate closing of research institutes capable of carrying out diagnostic work, and thus of identifying infected and, importantly for frontline health professionals, non‐infected individuals.

- *Rapidly developing pandemics necessitate rapid responses. Getting diagnostics and testing facilities up and running that are able to handle large numbers of samples are key to efforts to manage the disease. Crisis preparedness demands not only formulation of strategies to enable rapid scale up official facilities, but also advance identification of relevant available resources outside of the health system, and strategies of how to promptly and effectively harness them*.

Widespread testing for viral RNA must be complemented by widespread testing, or at least the testing of sentinels of the population, for anti‐viral antibodies. This is essential for relating the dynamics of infection to virus shedding (being infectious = infective for others) and to symptom development. And if (and this is a big IF) the presence of antiviral antibodies reflects protective immunity, antibody testing is essential for herd immunity policy scenarios, to provide the data needed for monitoring and modelling immune population densities/granularity, and also for identification of those in the population who are in principle protected and hence able to return to normality and spearhead safe exit strategies from lockdown measures.

- *Contingency planning requires identification of facilities or alliances able to promptly develop, produce in quantity and distribute easy‐to‐use antiviral antibody tests*.

### Treatment capacity

Patients having contracted COVID‐19 are grouped into three categories: in the China outbreak, 81% experienced mild, 14% severe and 5% critical infections (Wu and McGoogan, 2020). For those requiring hospitalization, and those with the most acute symptoms requiring intensive care, two key variables in treatment capacity seem to be bed availability generally, and in intensive care units particularly (images of patients lying on the floors of some hospitals made this abundantly clear to the entire world), on one hand, and ventilator/intubation tube availability for patients needing intubation, on the other. There are enormous differences between countries in terms the availability of hospital beds/1000 population (e.g., Japan 13.1, Germany 8.0, France 6.0, Switzerland 4.5, Italy 3.2, UK/Canada/Denmark 2.5, India 0.5: https://data.oecd.org/healtheqt/hospital‐beds.htm), ICU beds/1000 population, and numbers of available ventilators/intubation tubes (here, ventilators can be considered a proxy for any other clinical device that may be needed in a health crisis). An insufficiency of beds (and often health professionals) also resulted in ‘non‐critical’ interventions^2^ being postponed in some countries, and who, among critically ill patients suffering from different ailments, should be given an ICU bed. There are different reasons for insufficient beds and clinical devices. But the fact remains: some countries manage better than others; some countries do not have enough beds for even small increases representing normal fluctuations in patient needs and anticipated seasonal variations, let alone exceptional demands made by epidemics.

- *It is essential to increase bed, especially ICU bed, capacities in many countries, in order to reduce stress situations where patients cannot receive required treatment, and to prevent hospitals from being overwhelmed in times of crisis, when there is a spike in patients requiring hospitalization*.

One new development triggered by the COVID‐19 pandemic was the creation of so‐called ‘Fangcang shelter hospitals’—rapidly constructed, large scale, low cost, temporary hospitals, by converting existing public venues into healthcare facilities in Wuhan to manage the rapidly increasing COVID‐19 patient numbers (Chen *et al*., 2020). Instead of being delivered to regular hospitals, COVID‐19 patients were sent to shelter hospitals, where they were isolated and received basic medical care and frequent monitoring. Patients whose mild disease state transitioned to severe were then transferred to regular hospitals where they received intensive care. Although new emergency hospitals have been created in other countries, the purpose of these is generally to receive spillover from traditional hospitals, when these become overwhelmed by patient numbers. The purpose of Fangcang shelter hospitals is, in contrast, to centralize clinical management of the epi/pandemic *outside of the traditional hospital system*. COVID‐19 patients are channelled to shelter hospitals, thereby minimizing viral ingress into hospitals and infection of health professionals, and helping to maintain staffing levels and normal functioning of outpatient and inpatient facilities. It is important to note that shelter hospitals constitute low‐cost clinical settings—they require fewer health professionals and diagnostic‐treatment infrastructure than normal hospitals, because all patients have the same clinical issue and most have only mild‐to‐moderate disease—and thereby relieve pressure on traditional hospitals with sophisticated infrastructure in limited capacity that constitute high‐cost clinical settings (The creation of parallel low‐cost clinical settings to relieve pressure on limited capacity high cost clinical settings is more broadly applicable over and above epi/pandemic response situations: see Timmis and Timmis, 2017, for an example in primary healthcare).

- *Shelter hospitals should be incorporated into pandemic planning as the primary destination for pandemic patients, to allow traditional hospitals to continue functioning as normal as possible (or normally as long as possible)*.

### Protection of front‐line and ‘accessory’ front‐line professionals

It may seem so trivially obvious to say, but obviously needs saying because it was not apparent from the health system responses of a number of countries: those most at risk of infection are those in contact with the infected, i.e., front‐line doctors and nurses. And as they become infected, the numbers of available health professionals left to treat patients goes down as patient numbers go up. And, of course, infected health professionals become transmitters of infection among one another, and to uninfected patients, since in the hectic reality of emergencies, they may not always be able to practice adequate physical distancing. This obviously means that the greatest protection from infection must be accorded the front‐line professionals. However, there were substantive differences between countries in terms of the availability and use of best practice protective clothing (personal protective equipment, PPE) in the early days of the COVID‐19 crisis; these differences were mainly in different degrees of deficiencies. The incomplete protection of front‐line health professionals that occurred in a number of countries in the early days of the crisis, and that resulted in many infections and some deaths, is an unacceptable deficit in their health systems, particularly since the COVID‐19 outbreak was, from end of January 2020, a predictable disaster of international magnitude.

Then there are those one might designate *accessory* front‐line professionals: those who transport infected individuals, like ambulance drivers, non‐medical workers in hospitals, and so forth, carers ministering to people in care homes or in private homes, and others like some pharmacy and supermarket staff who, because of the nature of their work, come into physical contact with many people and cannot always achieve prescribed physical distancing. These are also particularly vulnerable to infection and to becoming infection transmitters. Since the people they care for are, because of their ages and underlying morbidities, often themselves particularly vulnerable to severe outcomes, infected carers may, unknowingly and unwillingly, become ‘angels of death’. Accessory front‐line professionals thus also require best PPE. There are wide regional and occupational differences in the availability and use of such clothing by these professionals.

In addition to the issue of PPE, there is the issue of hygiene in the workplace—the surfaces that become contaminated and sources of infection. While traditionally these have been cleaned by auxiliary staff, such people are themselves at considerable risk of being infected in such environments and, as a result, there may be an insufficient number to continue carrying out this task, thereby raising infection risk. Robots are in principle able to carry out various mechanical operations, so might take an increasing share in disinfection of high‐risk, high touch areas (e.g., robot‐controlled noncontact ultraviolet surface disinfection), and indeed other hospital tasks, such as delivering medications and food, diagnostic sample collection and transport, and so forth, (Yang *et al*., 2020), that may reduce both the work burden of overstretched staff and their infection risk.

- *Front‐line healthcare professionals and accessory front‐line professionals must be equipped with state‐of‐the‐art protection. In situations where the availability of such protection is limited, they must be prioritized for provision of best available protection*
- *The incorporation of robots into appropriate hospital operations should be energetically explored*

### Leadership, crisis management, international cooperation and coordination

Leadership in times of crisis is crucial to ongoing damage limitation and outcome severity, quite apart from its importance in planning crisis preparedness. Although we need to look back when all this is over, and take stock of what went right, what was wrong, and what went wrong (i.e., to perform a gap analysis), at this point it seems that most countries were on their own, acting largely independently of others during the SARS‐CoV‐2 outbreak, at least in the early days. However, a pandemic is by definition an international crisis, requiring an international response (national—self‐interest—policies may even be counterproductive in times of pandemics). Extensive and effective cooperation, coordination and sharing of resources were not evident (e.g., see Herszenhorn and Wheaton, 2020). Leadership quality and effectiveness varies significantly among countries and among relevant international agencies. Where leadership is suboptimal, dissemination of misinformation flourishes, and people are subjected to unnecessary levels of uncertainty and associated stress.

- *Effective and decisive, biomedical science‐guided, national and international leadership and coordination is absolutely crucial in pandemics, to prevent‐hinder‐manage‐minimize damage, acquire‐integrate‐learn from collective experience, make recommendations for crisis management, publish best practice procedures and standards. There is significant room for improvement*.

## Global preparedness–contingency planning

It is well known that experts have been warning of impending deadly epi/pandemics, including coronavirus outbreaks, for a long time (e.g., Turinici and Danchin, 2007; Ge *et al*., 2013; Menachery *et al*., 2015; https://www.ted.com/talks/bill_gates_the_next_outbreak_we_re_not_ready?language=en; Editorial (2016) Predicting pandemics, Lancet DOI: https://doi.org/10.1016/S0140-6736(16)32578-8 ; https://apps.who.int/gpmb/assets/annual_report/GPMB_annualreport_2019.pdf; https://www.weforum.org/agenda/2020/03/a-visual-history-of-pandemics/). Now while the nature, evolution, timing and source of novel emerging infectious agents is uncertain, pandemics are always counteracted by the same time‐honoured strategy: interruption of infection chains and anticipation of a surge in need for treatment of acute disease (here, we are nearly in the same situation as in the world confronted by Spanish flu in 1918). We, therefore, only need one epidemic preparedness. Despite this, the SARS‐CoV‐2 outbreak has clearly exposed how unprepared we were. There are multiple reasons for this, including.

### Contingency planning is long term, lacks immediacy and ‘wow factor’ and so may not always enjoy high political priority, and thus is often neglected

A primordial responsibility of government is to protect its citizens. This includes effective contingency planning for pandemics. However, due to the global nature of pandemics, coordination with neighbours (and factoring in potential flashpoints located more distantly, such as refugee camps in Greece and elsewhere, which could become, if not cared for, sources of a second wave of infection when the first is over), and intergovernmental cooperation is essential. Adequate contingency planning for deadly and devastating infectious disease outbreaks is not an optional policy, and the public have the right to insist on it, even if it becomes politically or economically expedient to neglect. For the public—the key stakeholders in this—transparency is essential and it must have access to information on the current state of preparedness, and future plans of government, and those of different political parties during election campaigns. Trusted biomedical science organizations must support the public in this by providing expert scrutiny and assessment. Governments must become accountable for the efforts they make to protect us.

- *Governments must engage the public in issues of crisis preparedness and publish their contingency plans for scrutiny*.
- *Scientific organizations should have press/web groups that become trusted sources for evidence‐based information for the public*.

### Catastrophe prediction/management expertise is not always at the heart of government, and thus able to inform and influence policy

Governments establish the presence of experts in key posts for topics they consider to be vital for informed policy and legislative activities. Such experts exert an influence in policy development by providing input that is up‐to‐the‐minute in a changing world. While some governments contain epidemic/catastrophe experts, others do not. Without such expertise, responses to catastrophes will generally be slow, *ad hoc* and inadequate, as has been the case in some countries in responding to the SARS‐CoV‐2 outbreak. For governments to fulfil their responsibilities to protect their citizens, it is essential that they have expert‐informed contingency planning. Learned societies and academies also have a major responsibility to seek to inform and influence government. The Royal Society, UK, and the American Society for Microbiology exemplify strategic influencing of national and international policy; other learned societies could be more pro‐active.

- *Expert scientists must be embedded in the heart of government to enable development of evidence‐based informed policies*

### Contingency planning is costly

Contingency planning involves *inter alia* the acquisition and maintenance of resources, such as beds, ICU capacity, stocks of ventilators, protective clothing, and so forth, in the case of pandemics (e.g., Kain and Fowler, 2019), that are *by definition* surplus to day‐to‐day requirements, and that will only be used if and when the catastrophe occurs. It also includes the development of generic platforms for rapid responses; in the case of pandemics, the development and testing of diagnostics, vaccine candidates, and effective treatments (see also below). This entails a significant recurring budgetary commitment. Political and economic viewpoints that such costs are not cost‐effective are fundamentally flawed because they generally only take into account the immediate cost elements, not the potential overall cost of the crisis and all its knock‐on effects. These are being brutally revealed by the unfolding SARS‐CoV‐2 outbreak which, at this still early stage, is involving governmental support of national economies amounting collectively to trillions of dollars. And this is only the tip of the economic iceberg. Bankruptcies, loss of employment, recession, loss of tax revenues, large scale deterioration of existing medical conditions in populations, potentially wide‐scale deterioration of mental health, and so forth, and the economic costs of these, also need to be taken into account when reflecting on the cost of the contingency planning insurance policy. As an illustration of knock‐on effects, global economic estimates of the benefits of vaccination have also shown that they extend well beyond those estimated from prevention of the specific disease in vaccinated individuals (Bloom, 2015). It is also worth comparing crisis preparedness costs with military expenditures. The latter are indeed budgetary commitments for preparedness for another type of crisis, namely a military conflict (excepting countries that use their military for internal affairs). And, as is the case in epi/pandemic preparedness, a considerable fraction of military resources is dedicated to surveillance operations. While accepting that military expenditures are also justified in terms of deterrence of hostile actions, and a multitude of non‐combat roles armed forces may undertake, it is not self‐evident that future military conflicts may result in losses of life and economic damage as high as the current COVID‐19 pandemic. In any case, in terms of protecting citizens, it should be abundantly clear that effective contingency preparedness for pandemics, and other crises,^3^ should be equated with military preparedness, and budgeted accordingly.

- *The principle of citizen protection demands that governments budget for adequate crisis preparedness in the same way that they budget for military preparedness. It is simply one of several essential ‘insurance premiums’ to which the state must commit*.

### Contingency planning also includes development and implementation of outbreak prevention strategies

From earlier infectious disease outbreaks, we can assume that the most probable source of a new pandemic will be an animal virus, probably a coronavirus, whose natural host is a wild animal, possibly a bat (e.g., see Brüssow, 2012), that mutates and, as a result, becomes infectious for humans, or for an intermediate host, from which it subsequently jumps to humans. Close contacts between humans and the animal host provide the opportunities for transmission. Reducing such close contacts will reduce the probability of spillover and thus of an outbreak. Close contacts between wild animals and humans occur in wet markets in Asia, small‐scale mixed farming activities with ducks and pigs, and so forth, or when humans encroach into wildlife habitats, e.g., through ecotourism, or destroy wildlife habitats for economic activities, forcing wildlife to enter human habitats (e.g., the destruction of rainforest for palm oil cultivation appears to have catalyzed a Nipah virus outbreak; Brüssow, 2012). In any case, although pathogen:host interactions underlying disease are generally well studied, current knowledge about the ecology of infectious agents – where pathogens are and what they are doing *prior to infection of humans*, especially those having alternative hosts, and how they are circulating and evolving new pathogenic and host‐range potential – is inadequate. In order to transit from response mode to pro‐active ecological measures to prevent outbreaks from occurring, there needs to be a major research effort to obtain a fundamental understanding of pathogen ecology (see e.g. Timmis, 2001).

- *Greater efforts are needed to reduce human:wildlife contacts and habitat overlaps, in order to decrease the probability of viral pandemics*
- *Effective outbreak prevention measures require acquisition of fundamental knowledge about pathogen ecology*

### Contingency planning and the public

#### Memory

It is human nature that, once this crisis is over, people, except those who lost loved ones, employment, and so forth, will generally want to forget it as quickly as possible and get back to normal. The number of individuals who try to keep it in the forefront of memory, in order to institute new measures that adequately protect us from the next crisis, *and there will undoubtedly be new crises* (see above), will be few and far between. Some, not all, leading politicians who now (often for the first time) insist that their responses are being guided by the best scientific evidence and advice, as though it were the most natural thing in the world, will quietly shed themselves of their scientific credentials and revert to business as usual, even when unpleasant issues like global warming, the antibiotic resistance crisis, our vulnerability to terrorist and cyber‐attacks,^4^ again come to the fore. In order that our collective memory retains the crucial need for crisis preparedness, it is essential that each year governments publish updated and independently audited contingency plans.

#### Literacy

And the public—the central stakeholders in, and funders of, government policy/actions—must be able to understand the issues and personally evaluate the sometimes vague policy statements they hear. To do this, society must become knowledgeable about/literate in such things. In the case of infectious disease crises, such as the one currently ravaging humanity, and the contingency plans necessary for these, literacy in relevant microbiology topics is, as we have previously argued, essential (Timmis *et al*., 2019).

#### Loss of civil liberties and public engagement

Interrupting the transmission chain in a pandemic may require lock‐down, which imposes major personal sacrifices on the public, including confinement: loss of freedom of movement/social activities/family visits; closure of workplaces/loss of employment and income, resulting in economic hardship/increases in debt; closure of schools/places of worship/hospitality venues/fitness studios/clubs of all sorts; restrictions on shopping; and elevated stress/worsening of psychiatric conditions. It is, therefore, crucial that such measures are accepted and supported by the public. For this, people must be engaged and presented with coherent lock‐down plans that are convincingly justified, in order to solicit compliance, solidarity and sharing of responsibilities. Federal structures, like those in the USA, Germany and Switzerland may lead to uncoordinated actions in different parts of the country that are unsettling and unconvincing, because the public perceives them as arbitrary. Such countries require coherent national plans that are consistent for the entire country.

Of course, all people in lock‐down want an exit as soon as possible, and it is essential for governments to develop and communicate as soon as possible their exit strategy, and the determining parameters and assumptions upon which it is based. Interestingly, some members of the public favour staggered exit plans, which implies a willingness to accept an infection risk. It will, therefore, be important for the government to have a public discussion on different risk scenarios, to obtain, present and discuss human/economic cost:benefit estimates (e.g., human lives against cost in loss of income /economic prosperity underlying the lock‐down versus herd immunity approach—how much unemployment averts how many deaths or years of productive life when considering the age structure of death). And this discussion needs to take place in the context of the probabilities of loss of life through other adverse causes, such as annual influenza epidemics.

#### Family‐friend contacts with terminally ill patients

One of the most shocking aspects of the COVID‐19 pandemic is the daily reporting of relatives of terminally ill patients who are unable to be with their loved ones at the end, and to pay last respects before burial. While this may be understandable in the context of patient isolation, social distancing, and the unbelievable hectic in overwhelmed ICUs, serious effort should be made to find a solution, perhaps moving terminally ill patients to an environment that permits both end‐of‐life patient care and limited safe contact with loved ones.

- *Governments must publish annual audited overviews of the national state of crisis preparedness, with critical analyses of its strengths and weaknesses and plans to address the weaknesses*
- *Governments and education ministries must raise public awareness of crisis potential and promote understanding of key elements of crisis management, inter alia through investing in school curricula changes and public information campaigns that increase literacy in topics such as microbiology and public health*
- *Governments should involve civil society in discussing restrictive measures because this increases compliance and the solidarity to shoulder the consequences*.

## Benchmarking

Achievement of optimal preparedness for, and operational responses to, a pandemic demands two things: international benchmarking and transparency/accountability in health systems (and of those who regulate and finance them). This includes chains of command and shared administrative responsibilities, procurement services, reliance on external suppliers of essential materials, and so forth. The disparities in responses we have listed above, that demonstrate significant differences between countries in the ability to respond to pandemics, are not justifiable in terms of operational efficiency, protection of front‐line professionals, clinical outcome, and so forth, and cannot be allowed to persist, to be manifested again in future crises. Health systems worldwide largely operate within narrow national perspectives, with little interest in better systems elsewhere. We urgently need objective and transparent benchmarking, and automatic mandating of adoption of the best practices in the world, where feasible. Transparency and convincing justification for failure to adopt the benchmark must become the norm. Of course, different health systems operate in different frameworks—payers, insurance, authorization and recommendation agencies, and so forth—but the tail cannot be allowed to wag the dog. Existing frameworks can no longer be accepted as default excuses not to improve. They must be adapted to allow adoption of the benchmarks, where possible, not the other way round. In the final analysis, there are only two elements relevant: the person in the ICU, who pays tax/insurance, and hence for the health system, and the government, which is responsible for health system functioning/evolution and protection of its citizens. Both of their goals are in principle aligned, so there should be no controversy: provision of the best achievable health system that is adequately prepared for catastrophic pandemics.

- *Governments and health systems must subject national health systems, and national health system crisis preparedness, to international benchmark scrutiny, and transparently strive for attainment of best international standards*.

## Improving diagnostic, prophylaxis and therapy capabilities

### Creating healthier pipelines of diagnostics and drugs

It is the responsibility of government to protect its citizens and the role of industry to innovate and create commercial products and services. These two goals are not always aligned for current clinical exigences. But to provide a vital health system, government and industry must align and form alliances that create synergies. There are, of course, many successful examples of such beneficial alliances. However, there is sometimes an unrealistic perception of the role of industry, particularly by some governments when confronted with a crisis for which they are not prepared, as articulated in the generic cry: why do not we have a vaccine for this, why do not we have a drug for that? For example, regulatory and payment hurdles incentivize industry to develop cancer drugs rather than antimicrobials, so it is irrational and unwarranted to complain about the poor state of pipelines for new antivirals in the time of COVID‐19, of antimicrobials in the time of the antimicrobial resistance crisis. If industry is to realign its research priorities towards current clinical priorities, it needs incentives to do so, e.g., through adequately funded creative government–industry–academia–clinical‐regulatory strategic alliances.

We have previously proposed a mechanism to create novel pipelines for accelerated discovery of new drugs and diagnostics (Timmis *et al*., 2014; and, simultaneously, to promote long‐term revival of struggling economies, interestingly in response to a financial crisis—that of 2008—which the SARS‐CoV‐2 pandemic will again unleash with considerable severity). This proposal calls for the use of infrastructure budgets (not overstretched research‐education‐health budgets) to be targeted to the creation of new strategic national/regional alliances between (i) cell biology and microbial diversity research groups, to discover and develop new diagnostics, drug targets and assays, and new drug leads from new microbes, (ii) biochemical engineers, chemists and pharma, to produce, evaluate and develop drug candidates, (iii) pharma, clinical research and regulatory agencies to assess clinical efficacy and safety of, and develop new drug candidates. In the context of the SARS‐CoV‐2 pandemic, an alliance between virology, cell biology, microbial diversity, and synthetic microbiology groups in upstream discovery would accelerate new antiviral discovery and populate antiviral drug pipelines, but also pipelines of new antimicrobials urgently needed for the treatment of bacterial superinfections responsible for some of the COVID‐19 mortalities. And: while advanced age, underlying co‐morbidities and infection dose are identified as predisposing factors for development of severe COVID‐19 disease, deaths among young healthy individuals also occur for reasons currently unknown. Once predisposing factors for this group have been elucidated, diagnostics to identify young people at risk, especially those most exposed to SARS‐CoV‐2, will be needed in order to reduce their exposure.

### Accelerating vaccine development

Vaccines, despite their proven value in protecting against disease and their much‐heralded pivotal importance for lockdown exit and herd immunity, are the Cinderellas of clinical practice and, in normal times, not only attract little interest from governments but also are controversial, due to negative publicity from vociferous anti‐vaccine groups propagating unfounded claims. The development and use of a number of current vaccines/vaccine candidates are orchestrated and funded, not by industry and public health systems, but by philanthropic organizations, like the Gates Foundation, working with agencies like CEPI (Coalition for Epidemic Preparedness Innovations) and GAVI (Global Alliance for Vaccines and Immunizations). Indeed, the Gates Foundation is also playing a leading role in the search for, and development of, a vaccine against COVID‐19 (https://www.gatesfoundation.org/Media-Center/Press-Releases/2020/02/Bill-and-Melinda-Gates-Foundation-Dedicates-Additional-Funding-to-the-Novel-Coronavirus-Response). However, it is the duty of national governments and international organizations, as part of their pandemic preparedness, to finance vaccine development platforms that are able to rapidly create new vaccines in response to an outbreak. Once a new vaccine candidate is shown to be safe and protective, its rapid large‐scale production and distribution requires the infrastructure of large pharmaceutical companies. As epidemics cannot be planned, industrial managers cannot be expected to promote projects without a market. Governments must therefore intervene to maintain the interest and technical capacity of industry in developing vaccines and antibiotics (a smouldering fire) by creating a market in form of governmental orders. Assessments of value‐for‐money of these strategic alliances must be made in the context of the global costs of pandemics like that of SARS‐CoV‐2.

- *Pandemic preparedness requires rapid creation, production and distribution of effective materials for diagnosis, prophylaxis and therapy. This necessitates significant long‐term investment in research and development involving unconventional alliances of disparate academic science and medical research groups, industry, philanthropic foundations, vaccine enabling coalitions, and crisis preparedness taskforces*.

## Increasing the preparedness and resilience of health systems

There is great diversity in stress resilience (e.g., the ability to deal with peaks of illness) of different health systems, with some being at least regionally overwhelmed during the winter influenza season. The less resilient systems will generally be the first to become overwhelmed in a health crisis. While there are numerous parameters involved in health system resilience, and experts know most of the pinch points and solutions that can deal with these (but also what is uncertain and what needs to be understood before effective ‘solutions’ can be formulated), three elements worth consideration in efforts to increase health system resilience are discussed here.

### Improving speed and coherence of responses to crises

Healthcare systems are by and large extremely large, complex, heavily bureaucratic and fragmented. The often system‐wide, multi‐level consultations, decisions and responses needed in times of emergencies are challenging and often slow, usually slower than crisis development, which means that healthcare systems follow and react to events, rather than managing them. Crises are in some ways analogous to wars, and bureaucracies are not designed to manage wars, which is the job of the military. In crises, we need crisis strategy‐tactics specialists, a taskforce with short, well defined and effective chains of command, tasked with overriding normal procedures and taking charge of supply chains and requisitioning of assets, (re)deployment of personnel, organization and prioritization of allocation of infrastructure, managing logistics, and so forth. These could be specially trained taskforces of existing staff within healthcare systems, external taskforces or combinations of both.

Of course, for taskforces to operate optimally, they, together with the best available scientific minds, must also plan in advance the required resources, supply chains, personnel, strategic options, and so forth. They must also organize regular ‘infection games’/public health manoeurvres (https://www.ted.com/talks/bill_gates_the_next_outbreak_we_re_not_ready?language=en) = crisis ‘fire drills’, to train nationally and transnationally, refresh skills and explore and anticipate unexpected events to ensure preparedness, so that appropriate responses can be rolled out rapidly anywhere, independently of national borders.^5^ Another, mandatory, task for the taskforce would be to conduct regular ‘stress tests’ of healthcare system resilience, as have been instituted for banks to ensure that they have adequate resources (= resilience) to withstand crises. Such stress tests should be designed by health experts, epidemiologists–modellers, procurement agencies, representatives of the diagnostics–vaccine–drug industry, and so forth, and the design and implementation of the stress tests overseen by the taskforce.

- *National crisis task forces consisting of dedicated strategy‐tactics specialists need to be established to plan crisis preparedness, make recommendations to improve health system resilience, and carry out regular crisis “fire drills” and stress tests*.

### Web‐based primary healthcare

An important aspect of the SARS‐CoV‐2 outbreak is that, in most countries, it has become more difficult to obtain consultations with primary healthcare clinics/physicians, because of social distancing practices, illness or involvement in crisis management (e.g., see Keesara *et al*., 2020). As time goes on, the inability to access many primary healthcare services leads to progressive worsening of existing and new conditions in some individuals. Access to primary healthcare, which in some countries was already unsatisfactory before Covid‐19, is becoming a new crisis. This has resulted in the ‘flight to the web’ for information (sometimes obtaining disinformation in the process): the web is becoming a substitute for clinical consultations, in terms of obtaining information relating to symptoms experienced. This will ultimately have a significant impact on how the public views the computer as a facilitator‐mediator of primary healthcare. While classical telemedicine—the *ad hoc* consultation of a remote, unknown physician who can advise on the symptoms presented—may be helpful in times of inadequate access to regular primary healthcare facilities, it cannot replace clinical advice informed by patient case histories and personal knowledge of the patient.

Reduced access to primary healthcare below a certain threshold constitutes itself a significant health hazard and is counter to a government's duty to protect its citizens. What to do to increase resilience of primary health care and increase access? One important contribution will be the ‘digital healthcare revolution’ (Keesara *et al*., 2020), i.e., some traditional one‐on‐one meetings between patient and doctor being replaced by web‐based consultations. But also imagine teleconsultations based on (i) complete personal case histories, combined with (ii) up‐to‐date population epidemiological information, combined with (iii) individual patient best practice recommendations based on precision medicine analyses/predictions: welcome to the National Clinical Informatics Centre (NCIC; Timmis and Timmis, 2017), informing in real time a *virtual doctor*, a clinically‐programed, AI‐evolving server. This *doctor*, interfacing with both the patient and NCIC, diagnoses according to detailed case history and patient symptom input via computer (and aided, where necessary, by diagnostic information obtained through in‐home patient self‐diagnosis with apparatus/diagnostic materials promptly delivered by a medical logistics service), and makes treatment recommendations (Timmis, 2020). In some countries/regions, access to primary healthcare already involves significant waiting periods. The additional restrictions on access to primary healthcare resulting from the SARS‐CoV‐2 outbreak are resulting in further suffering and frustration that will surely make the prospect of a consultation with a virtual doctor providing personalized medicine, who is instantly available 24/7, an increasingly attractive future possibility. Of course, many health issues cannot be handled remotely via the web (though the proportion will increase steadily with the development of informatic infrastructure and easy‐to‐use home diagnostics), and will result in referral to a clinician. But, web‐based consultations can significantly reduce numbers of patients requiring clinician consultations and the associated stress on the health system.

- *It is essential that health systems urgently develop centralized, secure informatic infrastructure needed to underpin web‐based machine learning‐facilitated precision medicine, and evolve web‐based consultations, available on demand 24/7, as an integral mainstream component of primary healthcare services*.

## Conclusions

The current SARS‐CoV‐2 outbreak has brutally exposed the current vulnerability of society to pandemics, even those that have been long predicted and anticipated (Ge *et al*., 2013; Menachery *et al*., 2015). Most healthcare systems have not evolved for resilience in times of catastrophe, nor for effective rapid responses to pandemics. A key principle steering evolution has been value‐for‐money within a fixed budget; contingency planning within this framework (outlays for materials that may never be used) may be considered to be a nuisance that diminishes what can otherwise be done with limited funds, and so to a greater or lesser extent may be postponed. For this reason, it is crucial that budgets for contingency planning are separate from health system budgets. Equally important, it has emphasized the fact that some healthcare systems have for a long time been on the edge of the cliff, just waiting for an event to push them over. Their adaptation to changing needs has often been through a ‘sticking plaster’ response. Evolution has been *ad hoc*, via responses to new developments and challenges, and often led to fragmentation rather than coherence. The lessons to be learned are thus not only to take scientifically‐founded pandemic predictions seriously into account in policy elaboration, but also to streamline and institute changes in healthcare systems that impose an evolutionary trajectory that increases coherence, efficiency and preparedness, and the necessary mechanisms to maintain these as new exigencies arise (e.g., see Timmis and Timmis, 2017). And, especially because this crisis has revealed enormous disparities in responsiveness, effectiveness and the quality of responses in different countries, both preparedness for pandemics and the general improvement of healthcare mandate international benchmarking for contingency planning and the evolution of healthcare systems. Comparisons/benchmarking within countries—within single systems—is no longer acceptable. Many healthcare systems need substantive improvements through strategic investments, in most cases targeted to system changes, not just extra funding of existing services. And above all, they need crisis taskforces embedded in them that can prepare for, and take charge in times of, impending catastrophes.

Another lesson learned is that the SARS‐CoV‐2 outbreak has revealed new synergy potentials, such as the manufacture of ventilators by engineering companies not normally active in the manufacture of medical devices. It is not unreasonable to assume that new innovations can and will emerge from new interactions between creative engineers and clinicians. For example, best practice for breathing difficulty and poor blood oxygenation is intubation and ventilation. The paucity of ventilators is a ‘critical control point’ for best treatment practice in some hospitals, which has been discussed above. Anecdotal evidence suggests that, of those individuals who die, despite best treatment practice involving intubation, the cause of death is often due to superinfection by antibiotic resistant bacteria (Vincent *et al*., 2020). The cause of this may indeed be intubation, causing perturbation of normal lung physiology and creating susceptibility to superinfection. There are, however, less invasive means of increasing blood oxygen levels. Perhaps engineers, together with clinicians, will devise new or improved non‐invasive approaches to blood oxygenation. And once creative engineers from the non‐medical field start to expertly scrutinize current medical devices, perhaps we will see new approaches and new designs that significantly advance medical practice.

But perhaps the most important lesson learned is about our frontline health professionals ministering to COVID‐19 patients, especially those with severe disease. These clinicians and nurses who willingly and selflessly work long, sometimes multiple shifts to the point of utter exhaustion, often not able to see their families for long periods because of the danger of infecting them, always under unbelievable stress working in what are essentially war zones with the accompanying horrors (e.g. see http://www.sixthtone.com/news/1005474/i‐spent‐seven‐weeks‐in‐a‐wuhan‐icu.‐heres‐what‐i‐learned?utm_source=sfmc&utm_medium=email&utm_campaign=2716680_Agenda_weekly‐17April2020&utm_term=&emailType=Newsletter), sometimes without adequate protective clothing and always in danger of contracting COVID‐19, sometimes becoming infected, and sometimes paying the ultimate price. These are the heroes of the pandemic, the faces of resilience of COVID‐19 healthcare, exceptional citizens demonstrating exceptional fortitude, personal sacrifice and professional dedication: they are our role models of the 21st century.

## Endnotes

1 However, note that considerable controversy exists about both numerator and denominator use, and indeed what represents a case, in the calculation of case fatality rates: Baud *et al*. (2020) estimate a 5.6% mortality rate in China, corrected for infection 14 days prior to death, whereas Spychalski *et al*. (2020) estimate 4.0% for the same population, when limited to closed cases.

2 In some hospitals, not only elective surgery is postponed, but many acute interventions are not made because the risk of COVID‐19 infection in hospital is too great

3 We need urgently epidemic prevention and preparedness policy as a part of the ecological crisis management at the global level, e.g. relocation plans for New York City, New Orleans, Venice, etc., in view of rising sea levels as a consequence of global warming.

4 For a rational discussion we need probability data on the likelihood of getting killed in a terrorist attack, by the current pandemic, by seasonal influenza, by sepsis by antibiotic‐resistant bacteria, to allocate financial resources in a way that the invested dollars have the greatest life‐saving effects.

5 One key thing the current crisis has taught us is that we are living in One‐World and not in the confines of national political organizations.
